# Nationalism and human rights: A replication and extension

**DOI:** 10.1371/journal.pone.0219409

**Published:** 2019-08-23

**Authors:** Joshua Holzer

**Affiliations:** Westminster College, Fulton, MO, United States of America; Valparaiso University, UNITED STATES

## Abstract

A recent article has found nationalism to be negatively associated with government respect for several human rights. In this article, I replicate the original study’s findings, I demonstrate that these findings are robust to an alternate model specification, and I then extend the analysis to additional human rights not examined by the original author. Ultimately, I find that in comparison to when the chief executive is *not* nationalist, when the chief executive is *highly* nationalist, that state is *less* likely to be associated with high government respect for six ‘empowerment’ rights (i.e. the freedoms of assembly and association, electoral self-determination, speech, foreign movement, religion, and worker’s rights), and *more* likely to be associated with low government respect for these six empowerment rights. This study suggests that nationalism’s influence on human rights is greater than previous thought.

## Introduction

In a recent article, Yazici ([1]: 147) “argue[s] that nationalism is inherently contradictory to human rights.” This is because nationalist political actors value “protecting national unity at any cost and prioritizing national interests over any other concerns,” which “jeopardize[s] certain types of human rights” (Yazici [1]: 147). In this article, I seek to take a closer look at the ‘certain types of human rights’ affected by nationalism. This article has four goals. First, I seek to replicate Yazici’s [1] original findings that the nationalism level of the chief executive is negatively associated with three different human rights: the freedoms of assembly and association, electoral self-determination, and speech. Second, I demonstrate that Yazici’s [1] original findings are robust to an alternate model specification that is more appropriate when analyzing ordinal dependent variables. Third, I examine whether the nationalism level of the chief executive is negatively associated with four additional human rights that Yazici [1] did *not* examine: the freedoms of domestic movement, foreign movement, religion, and worker’s rights. Fourth, I leverage the power of Tomz, Wittenberg, and King’s [2] Clarify software package to report predicted probabilities of all seven human rights. Ultimately, I find that in comparison to when the chief executive is *not* nationalist, when the chief executive is *highly* nationalist, that state is *less* likely to be associated with high government respect for six (of the seven examined) human rights (i.e. the freedoms of assembly and association, electoral self-determination, speech, foreign movement, religion, and worker’s rights), and *more* likely to be associated with low government respect for these six human rights. This study builds upon the findings of Yazici [1] and suggests that nationalism’s influence on human rights is greater than previous thought.

## Methods

### Sample

According to Yazici ([1]: 147), “democratic institutions…tame nationalism and diminish its effects on human rights.” However, ‘[i]n partial democracies, nationalist governments have access to means to violate human rights for the sake of national unity and security with less costs given unconsolidated democratic institutions” ([1]: 158). As such, for this article, I follow Yazici [1] in limiting my analyses to partial democracies. Like Yazici [1], I consult Marshall, Gurr, and Jaggers’ [3] Polity IV dataset and consider states to be partial democracies if they have a positive Polity score below 7.5. This follows Epstein et al. ([4]: 555) who suggests dividing Polity scores among the following three groupings: “Autocracies (Polity value −10 to 0), Partial Democracies (+1 to +7), and Full Democracies (+8 to +10).”

### Variables

For the dependent variables, I follow Yazici [1] in utilizing data from the Cingranelli-Richards (CIRI) Human Rights Dataset [5]. This dataset is comprised on two indices: the Physical Integrity Rights Index and the Empowerment Rights Index. Yazici ([1]: 155) notes that “physically violent acts draw more attention from media, become more visible, and bring immediate negative reactions at the domestic and international levels.” As such, he finds that nationalist chief executives in partial democracies do not excessively violate many physical integrity rights. However, he finds that nationalist chief executives *are* prone to violate certain empowerment rights as such rights “can be used by minorities to challenge the national unity” (Yazici [1]: 155). In order to examine the same rights as Yazici [1], I make use of CIRI’s Empowerment Rights Index, which includes data on the following rights: the freedoms of assembly and association, electoral self-determination, speech, domestic movement, foreign movement, religion, and finally worker’s rights [5]. CIRI scores for each of these rights range from ‘0’ (which indicates no respect) to ‘2’ (which indicates full respect). Note that for the remainder for this article, all references to ‘human rights’ specifically refer to the seven ‘empowerment rights’ measured by CIRI’s Empowerment Rights Index. Although for the sake of simplicity I effectively use the terms ‘human rights’ and ‘empowerment rights’ interchangeably, this does not imply that empowerment rights can be viewed as a proxy for all human rights, nor does it imply that the conclusions of this study extend to all human rights.

My primary independent variable is *nationalism level of the chief executive*, which is based on the primary independent variable used by Yazici [1]. According to Yazici ([1]: 152), “chief executives and their political parties are the main political actors responsible for human rights violations, and as such…it is plausible to hold these chief executives and their parties responsible for the human rights score of that year.” He argues that “by measuring these actors’ nationalism levels, we should be able to analyze their effect on human rights” Yazici ([1]: 152). In order to measure these actors’ nationalism levels, he utilized data from the Manifesto Project [6], which codes the manifests of political parties based upon fifty-six categories. Yazici ([1]: 152) then “use[d] three of these fifty-six categories to measure the nationalism levels of the chief executives’ political parties: positive mentions of national way of life, positive mentions of national security, and negative mentions of multiculturalism.” Finally, he averaged the scores from these three categories in order to construct his measure of nationalism, as he “consider[s] these categories as equally important pillars of nationalist ideology since they represent how much a political party is willing to sacrifice individual interests for the sake of national interests and to protect national unity” (Yazici [1]: 152). For this article, I have reconstructed Yazici’s [1] measure based upon the directions in his article using the same source material.

The control variables I include are also based upon those used by Yazci [1]. First, a measure of *conflict* drawn from Version 4-2014a of the UCDP/PRIO Armed Conflict database [7]. This variable is coded as ‘0’ for each country-year with less than 25 battle-related deaths and ‘1’ for each country-year with at least 25 battle-related deaths. Next, (logged) measures of population size and gross domestic product (GDP) per capita, both of which are from the World Bank [8]. Finally, Yazici ([1]: 7) argues that the “refugee population in a country may affect [the] level of nationalism…and human rights practices.” As such, like Yazici ([1]: 7), “I control for the effect of refugee flows in a given year by using a logged version of the ‘refugee population by country or territory of asylum’ variable from” the World Bank [8].

## Results and discussion

### Regression analysis

In Table 1, I present the results of three regressions that replicate the findings of Yazici [1]. Note that each model examines the *same* country-years and includes the *same* number of observations as the models used by Yazici [1]. As previously mentioned, to construct the *nationalism level of the chief executive* variable, I followed the directions outlined by Yazici [1] and consulted the *same* source material. Additionally, note that these models include the *same* control variables as was used by Yazici [1]. Finally, note that each regression is a panel-corrected standard errors (PCSE) model estimated using Stata’s xtpcse command with options to fix autocorrelation (i.e correlation(ar1)) and panel-level heteroskedasticity (i.e hetonly); this is the *same* model specification outlined by Yazici [1].

**Table 1:** PCSE estimates of CIRI scores in partial democracies.

	Model 1	Model 2	Model 3
	Assembly and association	Electoral self-determination	Speech
Nationalism level of the chief executive	-0.148***	-0.186***	-0.144***
	(0.050)	(0.038)	(0.044)
Conflict	-0.267*	-0.399***	-0.165
	(0.144)	(0.120)	(0.127)
(Logged) GDP per capita	0.162	0.333***	0.019
	(0.154)	(0.107)	(0.122)
(Logged) population size	-0.095	-0.105**	-0.103*
	(0.064)	(0.045)	(0.056)
(Logged) refugee population size	0.053*	-0.008	0.035*
	(0.030)	(0.025)	(0.021)
Constant	1.074	0.338	2.411**
	(1.377)	(1.024)	(1.031)
Observations	113	113	113

* *p* < 0.10,

** *p* < 0.05,

*** *p* < 0.01.

Figures in parentheses are heteroscedasticity-consistent standard errors. Note that higher values of the dependent variable indicate greater government respect for human rights.

Consistent with Yazici’s [1] results, Table 1 reports that the *nationalism level of the chief executive* is negatively associated with high CIRI scores for *assembly and association* (i.e. Model 1), *electoral self-determination* (i.e. Model 2), and *speech* (i.e. Model 3). For all three models, this relationship is statistically significant at the 99% level (which is, in fact, slightly better than the results reported by Yazici [1]). Also consistent with Yazici [1], all statistically significant control variables are pointing in the expected direction. For instance, *conflict* and *(logged) population size* are found to be negatively associated with high CIRI scores of the dependent variables, while *(logged) GDP per capita* and *(logged) refugee population size* are found to be positively associated with high CIRI scores of the dependent variables. I would like to point out that each variables’ coefficient differs slightly from those reported by Yazici [1]. This is likely the result of using different World Bank data, given that he cites the 2014 version of the World Development Indicators dataset, whereas I used the 2018 version of this dataset. Regardless, his coefficients and the coefficients from my replication models are nearly identical, which is a testament to the reproducibly of Yazici’s [1] original findings.

At this point, I would like to remind the reader that the dependent variables associated with each model in Table 1 all use an *ordinal* ‘0’ to ‘2’ scale. Long and Freese ([9]: 309) caution that while “it is tempting to analyze ordinal outcomes with the linear regression model (LRM)…an ordinal dependent variable violates the assumptions of LRM, which can lead to incorrect conclusions.” As such, “[w]ith ordinal outcomes, it is much better to use models that avoid the assumption that the distances between categories are equal” ([9]: 309). In other words, rather than using the PCSE model promoted by Yazici [1], Long and Freese ([9]: 309) point to McCullagh [10] who pioneered the use of ordered logistic regression. Indeed within the broader human rights literature, models that similarly use CIRI scores as the dependent variable seem to most commonly use ordered logit models (see for instance: [11–15]). As such, in Table 2, I present the results of three order logit regressions. Note that Models 4, 5, and 6 in Table 2 use the *same* variables and *same* sample as Models 1, 2, and 3 in Table 1. Again following the literature [16–21], also note that my standard errors are clustered by country. After having demonstrated that Yazici’s [1] findings are robust to independent verification, the goal now is to ascertain whether his findings are robust to an alternate specification.

**Table 2:** Ordered logit estimates of CIRI scores in partial democracies.

	Model 4	Model 5	Model 6
	Assembly and association	Electoral self-determination	Speech
Nationalism level of the chief executive	-0.478***	-0.803***	-0.705***
	(0.125)	(0.182)	(0.176)
Conflict	-1.902**	-2.058***	-2.048**
	(0.812)	(0.660)	(0.925)
(Logged) GDP per capita	0.915	1.419***	0.280
	(0.810)	(0.399)	(0.681)
(Logged) population size	-0.264	-0.396	-0.362
	(0.372)	(0.344)	(0.498)
(Logged) refugee population size	0.208	-0.043	0.248**
	(0.127)	(0.122)	(0.119)
Observations	113	113	113

* *p* < 0.10,

** *p* < 0.05,

*** *p* < 0.01.

Figures in parentheses are robust standard errors clustered by country. Note that higher values of the dependent variable indicate greater government respect for human rights.

As you can see, the results reported in Table 2 do not fundamentally differ from those reported in Table 1. The *nationalism level of the chief executive* is still negatively associated with high CIRI scores for *assembly and association* (i.e. Model 4), *electoral self-determination* (i.e. Model 5), and *speech* (i.e. Model 6). For all three models, this relationship is still statistically significant at the 99% level. Additionally, for all three models, all statistically significant control variables are still pointing in the expected direction.

As previously mentioned, CIRI’s Empowerment Rights Index includes data for *seven* different empowerment rights: assembly and association, electoral self-determination, speech, domestic movement, foreign movement, religion, and finally worker’s rights. Also as mentioned, Yazici [1] only examined nationalism’s effect on the first *three* of those rights. Given that it has been demonstrated that the nationalism level of the chief executive has a statistically significant effect on *each* of the three rights that have thus far been examined, it seems worthwhile to test whether the nationalism level of the chief executive similarly influences government respect for the remaining four remaining rights. As such, in Table 3, I present the results of four regressions that estimate the influence of the nationalism level of the chief executive on domestic movement, foreign movement, religion, and finally worker’s rights.

**Table 3:** Additional ordered logit estimates of CIRI scores in partial democracies.

	Model 7	Model 8	Model 9	Model 10
	Domestic movement	Foreign movement	Religion	Worker’s rights
Nationalism level of the chief executive	-0.260	-0.572***	-0.541**	-0.680***
	(0.198)	(0.198)	(0.272)	(0.212)
Conflict	-0.421	-1.222*	-1.386	-0.743
	(0.902)	(0.692)	(0.967)	(0.663)
(Logged) GDP per capita	0.428	0.662	0.608	0.306
	(0.755)	(0.684)	(0.492)	(0.667)
(Logged) population size	-0.057	-0.336	-0.296*	-0.658**
	(0.396)	(0.379)	(0.179)	(0.272)
(Logged) refugee population size	-0.090	0.17	-0.161	0.09
	0.165	(0.200)	(0.114)	(0.140)
Observations	113	113	113	113

* *p* < 0.10,

** *p* < 0.05,

*** *p* < 0.01.

Figures in parentheses are robust standard errors clustered by country. Note that higher values of the dependent variable indicate greater government respect for human rights.

As you can see, the *nationalism level of the chief executive* is negatively associated with high CIRI scores for *foreign movement* (i.e. Model 8), *religion* (i.e. Model 9), and finally *worker’s rights* (i.e. Model 10). For all three of these models, this relationship is statistically significant at least at the 95% level. For all three models, all statistically significant control variables are pointing in the expected direction. Notably, the *nationalism level of the chief executive* does not appear to influence government respect for the freedom to move around domestically (i.e. Model 7).

### Substantive effects

As mentioned above, a benefit of using ordered logit models over PCSE models when estimating CIRI scores (which are ordinal) is that you can avoid violating any LRM assumptions. Another benefit of using ordered logit models over PCSE models is that the substantive effects of the former can more easily be analyzed, as ordered logit models are supported in Tomz, Wittenberg, and King’s [2] Clarify software package (unlike PCSE models). Using this software package I can generate (and discuss) predicted probabilities in order to provide a more intuitive understanding of the relationships between nationalism and human rights.

In Table 4, I present predicted probabilities of CIRI scores—and *changes* in those predicted probabilities—when the *nationalism level of the chief executive* is at the minimum versus the maximum. Note that these probabilities are based upon each control variables’ mean (or mode in the case of *conflict*, given that this variable is categorical). Effectively, this means that the parameters used to generate these probabilities are based upon the ‘average’ state in my dataset, which has less than 25 battle-related deaths per year, a GDP per capita of roughly $7,700, a population of roughly 12 million, and a refugee population of about 3,600.

**Table 4:** The percentage change in predicted probabilities of CIRI scores when the nationalism level of the chief executive is at the minimum versus the maximum.

**Baseline probability when the nationalism level = min**			
	CIRI score		
	0	1	2
Assembly and association	0.055	0.394	0.552
	[0.023, 0.109]	[0.179, 0.616]	[0.289, 0.793]
Electoral self-determination	0.017	0.374	0.609
	[0.002, 0.060]	[0.147, 0.638]	[0.320, 0.848]
Speech	0.039	0.624	0.336
	[0.009, 0.109]	[0.241, 0.873]	[0.066, 0.740]
Domestic movement	0.035	0.273	0.692
	[0.002, 0.157]	[0.092, 0.519]	[0.414, 0.892]
Foreign movement	0.010	0.268	0.722
	[0.001, 0.050]	[0.066, 0.585]	[0.386, 0.932]
Religion	0.096	0.499	0.405
	[0.028, 0.230]	[0.327, 0.654]	[0.226, 0.606]
Worker’s rights	0.080	0.466	0.454
	[0.013, 0.254]	[0.297, 0.618]	[0.246, 0.674]
**Change (and % change) in baseline probability when the nationalism level → max**			
	CIRI score		
	0	1	2
Assembly and association	0.029	0.082	-0.111
	[0.137, 0.050]	[0.026, 0.139]	[-0.171, -0.052]
	53%	21%	-20%
Electoral self-determination	0.016	0.168	-0.184
	[0.004, 0.039]	[0.065, 0.244]	[-0.252, -0.092]
	95%	45%	-30%
Speech	0.032	0.093	-0.125
	[0.014, 0.058]	[-0.019, 0.219]	[-0.239, -0.023]
	81%	not significant	-37%
Domestic movement	0.005	0.047	-0.051
	[-0.012, 0.018]	[-0.015, 0.124]	[-0.130, 0.029]
	not significant	not significant	not significant
Foreign movement	0.006	0.103	-0.108
	[0.001, 0.021]	[0.035, 0.165]	[-0.170, -0.042]
	55%	38%	-15%
Religion	0.052	0.068	-0.120
	[0.001, 0.119]	[-0.004, 0.183]	[-0.250, -0.002]
	54%	not significant	-30%
Worker’s rights	0.051	0.102	-0.153
	[0.021, 0.088]	[-0.006, 0.233]	[-0.259, -0.051]
	64%	not significant	-34%

All probabilities were calculated based on the following parameters: < 25 battle-related deaths; GDP per capita of $7,749; population size of 12,311,939; and refugee population size of 3,605. 95% confidence intervals are in brackets. Note that higher CIRI scores indicate greater government respect for human rights.

Note that Table 4 is divided into two halves: the top half reports baseline probabilities when the *nationalism level of the chief executive* is at the minimum, while the bottom half reports changes (and percent changes) in each of the above baseline probabilities when the *nationalism level of the chief executive* goes from the minimum to the maximum. Starting with the top-right of the top-half portion, you can see that when the *nationalism level of the chief executive* is at the minimum, the probability of a CIRI score of ‘2’ for *assembly and association* is 0.552. This means that for a state that matches the ‘average’ state parameters, that state has a 0.552 probability of experiencing the highest level of government respect for the freedom of assembly and association. Moving now to the top-right of the bottom-half portion, you can see that when the *nationalism level of the chief executive* goes from the minimum to the maximum, the probability of a CIRI score of ‘2’ for *assembly and association* (i.e. 0.552) goes down by 0.111. While 0.111 may not seem like much, substantively, decreasing a 0.552 probability by 0.111 is actually a 20% decrease. This suggests that for a state that matches the ‘average’ state parameters, that state 20% *less* likely to experience the highest level of government respect for the freedom of assembly and association when the chief executive is *highly* nationalist (versus *not* nationalist).

Moving now to the top-left of the top-half portion, you can see that when the *nationalism level of the chief executive* is at the minimum, the probability of a CIRI score of ‘0’ (which indicates the lowest level of government respect for the freedom of assembly and association) is 0.055. Looking at the top-left of the bottom-half portion, you can see that when the *nationalism level of the chief executive* goes from the minimum to the maximum, the probability of a CIRI score of ‘0’ for *assembly and association* goes up by 0.029. Again, while 0.029 may not seem like much, substantively, increasing a 0.055 probability by 0.029 is actually a 53% increase. This suggests that for a state that matches the ‘average’ state parameters, that state 53% *more* likely to experience the lowest level of government respect for the freedom of assembly and association when the chief executive is *highly* nationalist (versus *not* nationalist). Note that this trend repeats itself for the freedoms of speech, foreign movement, religion, and finally worker’s rights. In each case, when the chief executive is *highly* nationalist (versus *not* nationalist) that state is *more* likely to experience the lowest level of government respect the the indicated human right, and *less* likely to experience the highest level of government respect for the indicated human right.

Lastly, I would like to direct the reader’s attention to the *domestic movement* row in the bottom-half portion of Table 4. As you can seen, I have indicated that each of the changes in the predicted probabilities is *not* significant; this is because each confidence interval overlaps with zero. Substantively, this suggests that chief executives that are *highly* nationalist do not appear to be any more likely to repress (or protect) the freedom of domestic movement, in comparison to chief executives that are *not* nationalist. Similarly, for several of the other rights, the ‘moderate’ level of government respect for that right (i.e. a CIRI score of ‘1’) is also not significant. This suggests that for this ‘moderate’ level of government respect, there is not a statistically significant difference between chief executives that are *highly* nationalist versus those that are *not* nationalist.

## Conclusion

In his recent paper, Yazici [1] examined nationalism’s impact on three of the seven rights in the CIRI Empowerment Rights Index. In this article, I have replicated Yazici’s [1] original models, I have provided an alternate (and more theoretically appropriate) specification of these models, and I have extended the analysis to the remaining four human rights that were not originally examined. Ultimately, I find that in comparison to when the chief executive is *not* nationalist, when the chief executive is *highly* nationalist, that state is *less* likely to be associated with high government respect for six (of the seven examined) human rights (i.e. the freedoms of assembly and association, electoral self-determination, speech, foreign movement, religion, and worker’s rights), and *more* likely to be associated with low government respect for these six human rights. Building upon Yazici’s ([1]: 147) original findings, this article further “help[s] scholars, politicians, and citizens better understand a potentially dangerous consequence of the rise of nationalism around the world.”
